# Effects of Color and Luminance Contrast on Size Perception—Evidence from a Horizontal Parallel Lines Illusion

**DOI:** 10.3390/vision2030028

**Published:** 2018-07-13

**Authors:** Xiaodan Zhang, Jiehui Qian, Qiaowei Liang, Zhengkang Huang

**Affiliations:** Department of Psychology, Sun Yat-Sen University, Guangzhou 510275, China

**Keywords:** perceptual illusion, color contrast, luminance contrast, sameness effect, depth perception

## Abstract

The present study investigated a size illusion composed of two horizontal lines that were vertically separated and parallel to each other. When the two lines were of equal length, the upper line was consistently perceived to be a little longer than the lower line, therefore it was termed as horizontal parallel lines (HPL) illusion. We investigated the effect of color and luminance contrast on the HPL illusion by manipulating the color and luminance of the two lines. Results indicated the following: (1) differences in color between the two lines reduced the illusion; (2) differences in luminance between the two lines reduced the illusion; (3) Effect 1 was greater than Effect 2; (4) the illusory effect could not be affected as long as both of the lines were of the same color or luminance. The results suggest that the color or luminance contrast may contribute to the overall decrease in the illusory effect for lines with different colors/luminances, but generally the illusion decreases as the two lines are less similar to each other. These findings indicate that the similarity or ‘sameness’ effect dominates the effect of color/luminance contrast on the size illusion over the effect resulted from contrast difference or depth perception.

## 1. Introduction

Perceptual illusions refer to distorted visual perception of an object or a figure that mismatches reality. For example, the Ponzo illusion, which is a renowned size illusion, refers to perceiving a distant object to be larger than a nearby object of the same physical size in an image. In addition to the Ponzo illusion, there are other well-known size illusions, like Muller–Lyer illusion, moon illusion, etc.

Explanations for these size illusions often involve in a compensation mechanism of size constancy for distance variation. Presumably, the phenomenon of size constancy demonstrates that the brain could account for an object’s angular size variation with viewing distance, since the apparent size of the object remains roughly constant regardless of distance. If an object’s angular size does not show the expected variation with the perceived distance, the brain infers that its physical size must be varying therefore its appearant size is compensated based on the perceived distance, resulting in size illusions like the Ponzo illusion. Indeed, research shows that a more realistic distance perception results in a stronger Ponzo illusion [1,2], supporting for this explanation. Similarly, the Muller–Lyer illusion can be explained by an effect of depth perception induced by the difference in orientations of the arrowheads of the two lines. When the arrowheads are pointing outwards from the line, the line is thought to be perceived as the corner of a room receding farther away than the line with the arrowheads pointing inwards, therefore the illusion occurs. It has been suggested that observers with no or little prior experience of modern architecture perceived significantly less amount of illusory effect than those with more experience [3], since the explanation of ‘misapplied size constancy’ usually relies on a top-down modulation of visual processing based on past experience [4].

On the other hand, research suggests that size illusions may also be resulted from interactions among local feature perception in a figure. For example, alternative explanations for the Ponzo illusion propose that the illusion is caused by the misperception of orientation induced by local visual cues (the ‘tilt constancy’ theory [5]); or by integrating the focal line lengths by various perceptual weights, which is determined by the relevance of local attentive fields (the ‘integrative field’ theory [6]). Another possible mechanism for the Muller–Lyer illusion is that since the overall length of the line with the arrowheads pointing outwards is longer than the line with the arrowheads pointing inwards, the perception of size is affected by the overall judgment and therefore the illusion occurs [7]. These explanations involve a bottom-up processing of local features in the figure, therefore, one might expect that features like color or luminance could affect such illusions. Indeed, studies show that color [8,9,10,11] and luminance [12,13,14] do play a role in size perception and size illusions.

In particular, studies on the Muller–Lyer illusion showed that differences in color [15,16,17] and luminance [8,15] between the arrowheads and the lines reduced the illusion. Researchers suggest that the smaller color/luminance difference is, the larger the illusory effect is, which is termed as the ‘sameness’ effect [12]. However, difference in color or luminance could as well serve as a depth cue and affect depth perception [18,19,20,21], which may further influence the size illusion. Due to the light-absorbing and light-scattering particles filled in the air, a distant object appears to be slightly grayer or less pronounced than a nearer object that is physically of the same color [18,22]. Conversely, depth perception can be induced by relative brightness or relative contrast, and the depth cue is called aerial perspective. Previous studies showed that reduced contrast was associated with judging an object as more distant [23,24,25,26,27,28]. Furthermore, research found that the perceived depth separation increased with increasing difference in brightness [19] and saturation [20]. For the Muller–Lyer illusion, depth perception deteriorates as the lines and arrows take on different colors or luminances and therefore become less similar, which coincided with the effect of ‘sameness’, and the two explanations could not be torn part. To our knowledge, the possible contribution of depth perception in the effect of color and luminance contrast on size illusions has not been investigated in the past literature. Therefore, it is unclear whether the effect of color or luminance on size illusions like Muller–Lyer is due to color/luminance difference per se (‘sameness’ effect) or an indirect influence from depth perception. In this study, we aimed to reinvestigate the effect of color and luminance on a simple size illusion and to clarify the possible mechanisms underlying this effect.

**Figure 1:**
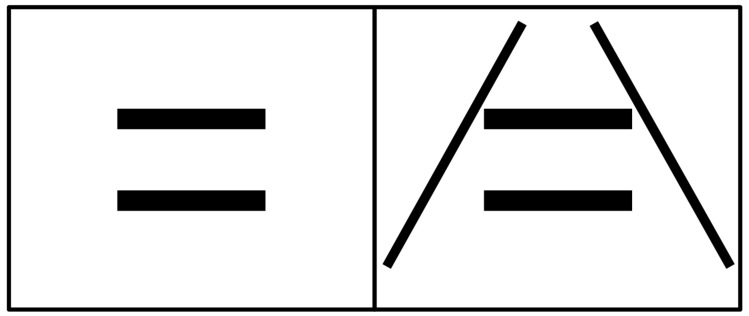
Size illusions. **Left panel:** Horizontal parallel lines (HPL) illusion. When the two lines were of equal length, the upper line was consistently perceived to be a little longer than the lower line. **Right panel:** a simplified version of the Ponzo illusion.

The size illusion is composed of two horizontal lines that are vertically separated and parallel to each other. When the two lines were of equal length, the upper line was consistently perceived to be a little longer than the lower line. This illusion is termed as horizontal parallel lines (HPL) illusion [29] (Figure 1, the left panel). It can be reliably observed, although the reported illusory effect was about 2% [29], which is quite small compared to the well-known Ponzo or the Muller–Lyer illusions that shows a typical 10–30% illusory effect [2,15]. The mechanism underlying the HPL illusion is unclear. Because the configuration of the illusion is akin to the Ponzo illusion (a simplified version of the Ponzo illusion is shown in Figure 1, the right panel), one might suspect a ‘misapplied size constancy’ mechanism to explain the phenomenon. That is, people tend to perceive the upper line as being farther away and the lower one as being closer to us, and therefore more compensation for distance is applied to the upper line than to the lower line that has the same angular size, resulting in the illusion. However, since the key visual components that induce depth perception in the Ponzo illusion (e.g., linear perspective cue) are lacking in the HPL illusion, it is also possible that other mechanisms may contribute to the illusion.

By employing the Horizontal parallel lines illusion, we investigated the effects of color and luminance difference on size perception. Experiment 1 replicated the previous study [29] that the illusion exists for lines of the same color; Experiment 2 examined the effect of luminance by testing lines of the same grayness; Experiments 3–5 examined the illusion by setting the two lines to different colors and luminances. Two possible explanations of the effect of color and luminance difference—an indirect contribution of depth perception or the ‘sameness’ effect—were contrasted to examine the underlying mechanisms of the effect of color and luminance on size illusions.

## 2. Methods

### 2.1. Participants

Sixteen students from Sun Yat-Sen University with normal or corrected-to-normal vision took part in Experiment 1 for pay, 15 took part in Experiment 2, 15 students took part in Experiment 3, and another 19 took part in Experiments 4 and 5. All of them were naive to the purpose of the study. This research was approved by the Sun Yat-Sen University Institutional Review Board (IRB). Written informed consent was obtained from each participant prior to each experiment.

### 2.2. Apparatus

The experimental task and stimuli were generated with MATLAB using the Psychophysics Toolbox 3.0 extension [30,31] and were displayed against a uniform white background on a 23-inch HP proDisplay P231 monitor. The display resolution was set to 1920 × 1080, with a refresh rate of 60 Hz. For the typical viewing distance of 70 cm, a pixel subtended approximately 1 arcmin.

### 2.3. Materials

The stimuli were composed of two horizontal parallel lines (Figure 1). During the experiment, one of the lines was fixed at $5^{\circ }$ with a width of $0.5^{\circ }$, while the other line varied in length in order to measure the illusory effect. The vertical separation between two line was of $3.5^{\circ }$.

**Figure 2:**
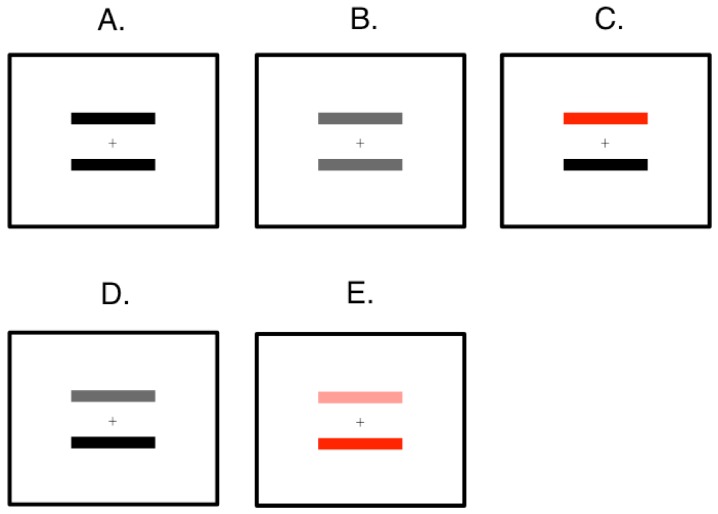
Exemplar stimuli in the experiments: Experiment 1 (**A**); Experiment 2 (**B**); Experiment 3 (**C**); Experiment 4 (**D**) and Experiment 5 (**E**).

Different colors were applied to the two lines in each experiment (see Table 1 for their luminances, Michelson contrast: $\frac{L_{max}-L_{min}}{L_{max}+L_{min}}$, and XYZ values in CIE 1931 color space). Four colors were used in the study: black, blue, red, and, green. In addition, different shades of gray were also used, where ‘grayB’, ‘grayR’ and ‘grayG’ had the same luminance values as blue, red and green, respectively. In this article, an abbreviation of ‘color1–color2’ was used as a convention to denote for a certain color combination in an experiment, where *color1* indicated the color of the upper line and *color2* indicated the color of the lower line. For example, a color combination of red–green suggested that the upper line was of red and the lower line was of green. In Experiment 1, we tested the following color combinations: black–black, blue–blue, red–red, and green–green; in Experiment 2, we tested grayB–grayB, grayR–grayR, and grayG–grayG; in Experiment 3, we tested blue–black, red–black, and green–black; in Experiment 4, we tested grayB–black, grayR–black, and grayG–black; in Experiment 5, we tested light red–red. Exemplar stimuli were shown in Figure 2.

### 2.4. Procedure

Observers were seated in a dark room to complete the experiments. Ten practice trials were given for them to get acquainted with the stimuli and the task. Each trial began with a fixation cross that subtended $0.3^{\circ }\times 0.3^{\circ }$ shown at the center of the screen and remained throughout the trial. The stimuli of the HPL were then presented for 1500 ms, followed by a blank screen until observers responded. The observers were instructed to judge whether the upper line or the lower line was perceived to be longer while keeping their fixation at the center of the screen. If the upper line was perceived to be longer, they were requested to press the key “1”, otherwise, press the key “2”. For a subset of the participants, the response keys were switched, i.e., pressing key “1” indicated the upper line being perceived as shorter, and vice versa. If no difference was perceived between the upper and the lower lines, they were instructed to equally distribute ‘no difference’ answer between the “1” and the “2” keys. We encouraged them to discriminate even a tiny slight difference between the two lines, and to try their best to avoid ‘no difference’ answer. The response and reaction time of each observer were recorded. After the response, an 1000 ms blank intertrial interval was presented before the next trial.

One of the lines varied in length to measure the illusory effect. Some participants were tested on trials with the upper line varied; others were tested on trials with the lower line varied. The levels of physical length tested were shown in Table 2. Since the reported illusory effect was about 2% [29], most trials were tested on a range of upper line length between $4.90^{\circ }$ and $5.00^{\circ }$ with the length of lower line fixed, or on a range of lower line length between $5.00^{\circ }$ and $5.10^{\circ }$ with the length of upper line fixed. Each participant completed 83 trials for each color combination. The order of all trials was randomized. The participants rested for 10 s for every 100 trials.

### 2.5. Data Analysis

For preliminary data screening, we excluded the participants whose data showed extreme reaction times, that was, beyond two standard deviations from the mean or less than 200 ms. Through this approach, 4 participants were excluded in Experiment 1, 4 in Experiment 2, 2 in Experiment 3, and 2 in Experiments 4 and 5.

The effect of the illusion was defined as the percentage of increase in the perceived upper line length. To compensate for the illusory effect, the physical length of the upper line had to decrease (or the physical length of the lower line had to increase) by the same amount in order for observers to perceive the upper and the lower lines as equal. For data analysis, the probability of the upper line being perceived as longer was plotted against the percent of physical decrease of the upper line length (or the percent of physical increase of the lower line length). The data was fitted with a Weibull function. The illusory effect was calculated as the point of subjective equality (PSE), which was derived as the percent of physical decrease of the upper line length (or the percent of physical increase of the lower line length) that corresponded to a 50% probability on the Weibull curve. A PSE greater than zero indicates an overestimation of the upper line length and a PSE less than zero indicates the opposite.

## 3. Results

### 3.1. Experiment 1

It is controversial whether color could influence the magnitude of the size illusion. While research on the Muller–Lyer illusion shows that applying different colors to the whole stimuli (e.g., red Muller–Lyer stimuli or green Muller–Lyer stimuli) could result in differences in the magnitude of the illusion [11,12], no such effect was found for the HPL illusion [29]. Therefore, the purpose of this experiment was to replicate Zhao et al.’s findings and to test whether the HPL illusion occurred with the upper and the lower lines being of the same color (one-color stimuli).

**Figure 3:**
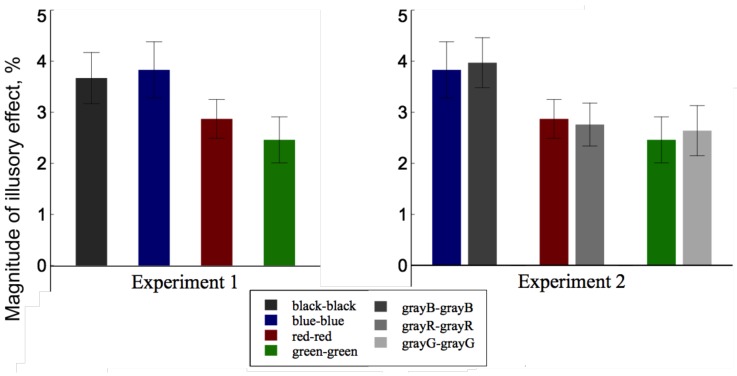
Results of Experiments 1–2. **Left panel:** results of Experiment 1; **right panel:** comparison between the results of Experiments 1 and 2. Error bar represents one standard error.

The illusory effects for different color combinations varied from 2.46% to 3.83% (Figure 3, the left panel). In other words, the physical length of the upper line had to be decreased by about 0.12–0.18${}^{\circ }$ in order for the observers to perceive the two lines as equal. An one-sample *t* test was performed for each color combination to examine the significancy of the illusion. The magnitudes of the illusory effect were significantly different from zero (zero indicates no effect) for black–black, [t(15)=6.19,p<0.001, *d* = 3.20], blue–blue, [t(15)=6.56,p<0.001,d=3.38], red–red, [t(15)=7.46,p<0.001,d=3.85], and green–green, [t(15)=5.45,p<0.001,d=2.81]. Repeated-measures analysis of variance (ANOVA) showed that there was no significant difference between the four color combinations, [$F\left(3,45\right)=0.14,p=0.92,\eta_{p}^{2}=0.001$].

### 3.2. Experiment 2

Past research suggests that the effect of color on the Muller–Lyer illusion may be attribute to a change in luminance contrast between the stimuli and the background [11,32]. However, it is unknown whether this account could be generalized to the HPL illusion given that the effect of color for one-color stimuli was not generalized to the HPL illusion. In Experiment 2, we examined whether the illusory effect changes with the luminance contrast between the one-color (gray) stimuli and the background.

The illusory effects for different gray combinations varied from 2.64% to 3.97% (Figure 3, the right panel). In other words, the physical length of the upper line had to be decreased by about $0.13^{\circ }$ to $0.19^{\circ }$ in order for the observers to perceive the two lines as equal. An one-sample *t* test was performed for each gray combination to examine the significancy of the illusion. The magnitudes of the illusory effect were significantly different from zero for grayB–grayB, [t(14)=6.79,p<0.001,d=3.62], grayR–grayR, [t(14)=5.60,p<0.001,d=2.99], and for grayG–grayG, [t(14)=5.49,p<0.001,d=2.93].

Compared with the results of blue–blue lines, red–red lines and green–green lines in Experiment 1, a 2 × 3 (experiment × luminance) mixed-design ANOVA showed that there was no significant difference between color combinations (Experiment 1) and gray combinations (Experiment 2), [$F\left(1,29\right)=0.25,p=0.62,\eta_{p}^{2}=0.02$], and no significant difference between blue/grayB, red/grayR and green/grayG combinations, [$F\left(2,58\right)=0.46,p=0.64,\eta_{p}^{2}=0.08$]. No interaction was found.

### 3.3. Experiment 3

Although disputable [11,32,33], several studies demonstrated that the size illusion was larger with smaller color difference within the stimuli [8,12,34]. In this experiment, we employed the lines with different color–black combinations (two-color stimuli) to investigate whether color contrast in the stimuli could affect the illusion.

**Figure 4:**
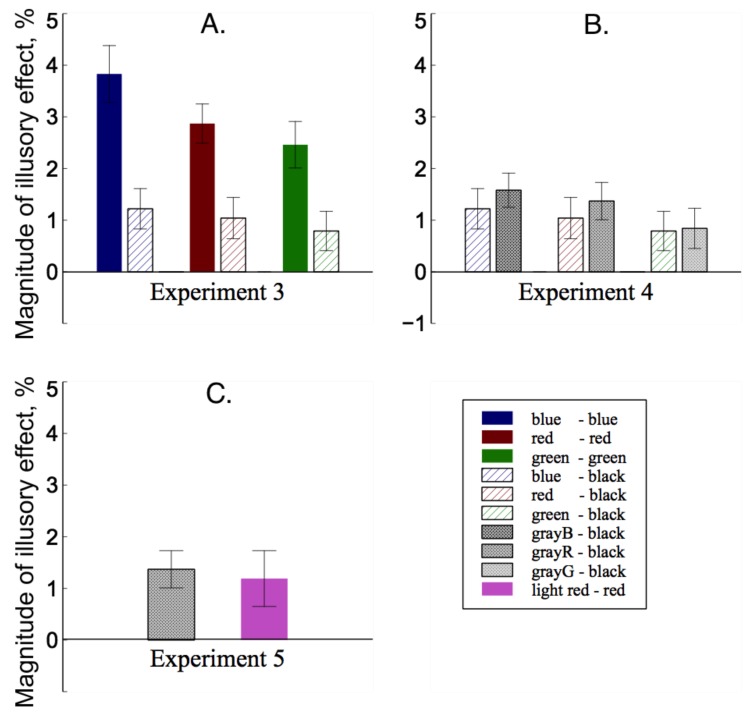
Results of Experiments 3–5. (**A**) comparison between the results of Experiments 1 and 3; (**B**) comparison between the results of Experiments 3 and 4; (**C**) comparison between the results of Experiments 4 and 5. Error bar represents one standard error.

The illusory effects for different color–black combinations varied from 0.79% to 1.22% (Figure 4A). In other words, the physical length of the upper line had to be decreased by about $0.04^{\circ }$ to $0.06^{\circ }$ in order for the observers to perceive the two lines as equal. The illusory effects for blue–black and red–black combinations were significantly different from zero: blue–black, [t(14)=2.79,p=0.016,d=1.49]; red–black, [t(14)=2.63,p=0.020,d=1.40]; However, the effect for green–black combinations was not, [t(14)=1.85,p=0.085,d=0.99].

Compared with the results of blue–blue lines, red–red lines and green–green lines in Experiment 1, a 2 × 3 (experiment × color) mixed design ANOVA showed that the illusory effect was significantly greater for the two lines consisting of same color, [$F\left(1,29\right)=12.85,p=0.001,\eta_{p}^{2}=0.31$]. There were significant differences between blue, red and green combinations, [$F\left(2,58\right)=8.77,p\leq 0.001,\eta_{p}^{2}=0.23$]. Post-hoc test showed that green combinations were significantly lower than the blue combinations, p=0.004 (Bonferroni corrected) and the red combinations, p=0.007 (Bonferroni corrected). There was no significant interaction found, [$F\left(2,58\right)=2.04,p=0.06,\eta_{p}^{2}=0.09$].

### 3.4. Experiment 4

In Experiment 4, we tested the effect of luminance by employing stimuli with the upper and the lower lines being both of gray but differed in luminance (two-tone stimuli). In this way, we could investigate whether the color effect found in Experiment 3 was caused by differences in color contrast or luminance contrast. In addition, since past studies on the effect of color/luminance did not discriminate the contribution of depth perception and similarity (‘sameness’ explanation), here we attempted to distinguish the two explanations by lowering the luminance contrast between the upper line and the background. The depth perception explanation predicted a stronger size illusion as the upper line with lower contrast could be perceived to be more distant, while the ‘sameness’ explanation predicted a weaker illusion as the upper and lower lines became less similar. By this manipulation, we could clarify the mechanism underlying the effect of luminance contrast on size illusion.

The illusory effects varied from 0.84% to 1.58% (Figure 4B). In other words, the physical length of the upper line had to be decreased by about $0.04^{\circ }$ to $0.08^{\circ }$ in order for the observers to perceive the two lines as equal. The illusory effects for grayB–black and grayR–black combinations were significantly different from zero: grayB–black, [t(18)=3.25,p=0.004,d=1.73]; grayR–black, [t(18)=2.38,p=0.028,d=1.47]; However, the effect for grayG–black combinations was not, [t(18)=0.17,p=0.87,d=0.09].

Compared with the results of blue–black lines, red–black lines and green–black lines in Experiment 3, a 2 × 3 (experiment × color) mixed design ANOVA showed that the illusory effect in Experiment 4 was significantly greater than that in Experiment 3, [$F\left(1,32\right)=6.32,p=0.017,\eta_{p}^{2}=0.13$]. There were significant differences between blue, red and green combinations, [$F\left(2,64\right)=0.67,p=0.005,\eta_{p}^{2}=0.22$]. Post-hoc test showed that green combinations were significantly lower than the blue combinations, p=0.034 (Bonferroni corrected) and the red combinations, p=0.047 (Bonferroni corrected). There was no significant interaction found, [$F\left(2,64\right)=0.86,p=0.92,\eta_{p}^{2}=0.003$].

### 3.5. Experiment 5

Experiment 4 found that the illusory effect for the two-tone stimuli was significantly greater than that for the two-color stimuli. However, since research shows that processing for color and luminance involves relatively independent functional subdivisions in the visual system [35,36], we may respond differently to stimuli consisted of various grayness and stimuli consisted of colors with the same hue but various luminance. Therefore, in Experiment 5, we employed stimuli that were same in hue but different in luminance to confirm the findings in Experiment 4.

The mean illusory effect for light red–red lines was 1.19% (Figure 4C). The magnitude of the illusory effect was significantly different from zero, [t(18)=5.12,p=0.036,d=2.41]. Compared with the result of grayR–black lines in Experiment 4, a paired-sample *t* test showed that the illusory effect was not significantly different between the two combinations, [t(18)=1.15,p=0.26,d=0.61].

## 4. Discussion

The present study investigated the effect of color and luminance on a size illusion—Horizontal parallel lines illusion. Experiments 1 and 2 showed that the HPL illusion occurred with one-color stimuli, and there is no significant difference in the illusory effect between stimuli with different colors or different luminances. Experiments 3 and 4 demonstrated that the illusory effect diminished when the upper and the lower lines consisted of different colors or grayness, but the illusion was stronger for two-tone stimuli than two-color stimuli. Experiment 5 confirmed the results of Experiment 4 by employing stimuli with the same hue but different luminances.

Our results showed that neither color nor luminance affect the HPL illusion as long as the two lines were of the same color and luminance. Although there was a trend of decrease in the illusory effect as the luminance contrast between the lines and the background decreased and the mean illusory effect for the blue and red stimuli was greater than that for the green stimuli (Experiments 1 and 2), which was consistent with the previous studies [15,37], the difference was not significant. The lack of an effect of color and luminance contradicts the findings on several other size illusions [11,15,34]. This is possibly due to the relatively small illusory effect for the HPL illusion, compared to the well-known Ponzo illusion or the Muller–Lyer ilusion [2,15]. Since the full illusion is small, variations in the illusory effect resulted from general color or luminance contrast between the figure and the backgrond can be trivial and therefore to detect a significant change in the illusory effect may require a much larger sample size. Another possibility is that the difference in luminance contrast among the blue, red and green used in our study was too small to result in a significant change in the illusory effect. Indeed, Sadza and de Weert found that a luminance ratio of 2 between the two stimuli did not cause a significant effect in the Muller–Lyer illusion, whereas a factor of 4 demonstrated the effect of luminance difference [15]. This was also consistent with Bulter’s findings [38]. In our study, the luminance ratio was less than a factor of 4 between any two color stimuli and their corresponding gray stimuli, it is possible that such a luminance difference is not sufficient to induce significant changes in the illusion.

Experiment 3 showed that color differences between the upper and the lower lines significantly affected the illusion, consistent with previous findings [11,12]. There was a clear decrease in the illusory effect for the two-color stimuli, especially for the green–black combination. Experiment 4 replicated the results of Experiment 3 using two-tone stimuli, which suggests that the effect of color difference might also be attributed to luminance difference between the upper and lower lines. In addition, the illusory effect for lines with gray–black combinations were significantly greater than that for lines with color–black combinations. Note that the colors used in our experiments had equivalent luminance values with the corresponding grays, suggesting that the luminance difference between the two lines cannot sufficiently explain this effect. Since the appearance of gray is more similar to black than other colors, the difference between the effect of color (Experiment 3) and the effect of luminance (Experiment 4) may be attributed to a ‘sameness’ explanation. Compared with the results of Experiments 1 and 2, we found that the greatest illusory effects are evoked by a figure in which the lines are most similar to each other. A Gestalt principle of similarity grouping indicates that objects that are similar to each other tend to be perceived as a whole [39]. It is possible that a perception of wholeness facilitates the size comparison process, therefore the illusion is greater with more similar components in the figure.

In contrary, the results of Experiments 4 and 5 could not support the explanation that the effect of luminance contrast on the size illusion is due to reduced depth perception. The aerial perspective cue demonstrates that a distant object appears to be fainter than a nearer object with the same color [22], and conversely, reduced luminance contrast is associated with judging an object as more distant [23,24,26,27,28]. According to this account, the upper line consisting of gray in Experiment 4, and that of light red in Experiment 5, should be judged as more distant, because they had lower contrast in luminance and saturation with the background compared to the lower line. Since research shows that more realistic depth perception could result in a stronger size illusion [1], introducing the luminance contrast in the stimuli of Experiments 4 and 5 would enhance the depth perception in the figure, which predicted a stronger size illusion to be observed. Since the results contradict this prediction, we conclude that the contribution of depth perception in the effect of color/luminance difference, if any, is much smaller than the contribution of ‘sameness’ effect. Overall, our results suggest that the ‘sameness’ effect dominates the effect of color and luminance difference on the size illusion.

The mechanisms underlying the HPL illusion is still unknown. Because of its relatively small illusory effect, the illusion is seldomly reported in the literature. As many of the size illusions like Ponzo or Muller–Lyer, the ‘misapplied size constancy’ mechanism might be reasonable to explain the phenomenon. However, since there is no explicit depth cues present in the HPL configuration, the generation of depth perception might involve certain ‘hidden assumptions’ based on past experience [40]. For example, assumptions on the location of the horizon could trigger depth cues like horizon ratio or height in the visual field, as both of these cues rely on knowing where the horizon is. Horizon ratio suggests that an object closer to the horizon is perceived to be more distant; and height in the visual field suggests that an object that rest on a surface below the horizon and is higher in the field of view is usually seen as being more distant [41,42,43]. If we assume that the location of the horizon was at the upper field of view, then the upper line would be perceived to be more distant than the lower line, and the illusion can be induced. However, unlike the Ponzo illusion where the two tilted lines resembling railroad tracks (Figure 1, the right panel) provide solid basis for such assumption, it seems more liberal for an observer to assume where the horizon is in the HPL illusion. In order to test whether there is a ‘hidden assumption’ for the location of horizon, we performed a control experiment in which 10 participants were asked to indicate the location of horizon on a blank white screen with a fixation cross placed at the center of the display. Seven out of 10 participants indicated that the horizon was at the upper field of view, although the exact locations varied across participants. This suggests that it is possible that the ‘misapplied size constancy’ hypothesis can be accounted for the HPL illusion, at least for those who assume that the horizon is located at the upper field of view.

Besides the ‘misapplied size constancy’ hypothesis, we also suspect that the illusion occurs because of an uneven distribution of mental efforts when scanning and processing the upper and the lower lines. Research suggests that attention sequentially shifts from left to right and from top to bottom when reading or scanning information [44,45]. A glance at the figure may signify the visual system that the two lines are similar, as a result, greater mental efforts and more cognitive resources may be attributed to the upper line for accurately perceiving its size and fewer to the lower line as its similarity to the former, resulting in perceiving the upper line as a little longer than the lower line. With this explanation, we predict that the Horizontal parallel lines illusion could occur in a population with a reading habit of horizontally scanning the text line by line from top to bottom, and a similar Vertical parallel lines illusion (left line perceived to be longer) could occur in a population with a reading habit of vertically scanning the text line by line from left to right. However, due to the lack of suitable participants, the latter prediction cannot be tested in our study. Future eye tracking evidence may shed light on the possible explanations for the illusion.

## 5. Conclusions

We investigated the effect of color and luminance contrast on size perception using a size illusion of horizontal parallel lines. Applying different colors or luminances to one-color stimuli does not seem to affect the illusion, but creating color or luminance differences between the upper and the lower lines decreases and even diminishes the illusion. The color or luminance contrast may contribute to the overall decrease in the illusion for lines with different colors/luminances, but generally, the illusion decreases significantly as the two lines are less similar to each other. Therefore, our results support a ‘sameness’ explanation to the effect of color/luminance contrast on size illusions like HPL.

## Figures and Tables

**Table 1:** Colors used in the study and their luminances (cd/m${}^{2}$), Michelson contrasts, and XYZ values in CIE 1931 color space.

Color	Black	Blue	Red	Green	Light Red
Luminance	0.12	25.8	34.6	96.8	51.5
Michelson contrast	0.99	0.65	0.55	0.11	0.40
CIE XYZ	[0.95, 1, 1.08]	[0.77, 0.92, 0.13]	[0.53, 0.78, 1.06]	[0.59, 0.28, 0.96]	[0.15, 0.22, 0.30]
**Color**	**White**	**grayB**	**grayR**	**grayG**	
Luminance	120.1	25.5	34.2	96.5	
Michelson contrast	0	0.65	0.55	0.11
CIE XYZ	[0, 0, 0]	[0.72, 0.76, 0.83]	[0.17, 0.18, 0.20]	[0.06, 0.06, 0.07]	

**Table 2:** Line lengths tested in the study.

*Lower line fixed*									
Upper line length	$4.8^{\circ }$	$4.85^{\circ }$	$4.90^{\circ }$	$4.925^{\circ }$	$4.95^{\circ }$	$4.975^{\circ }$	$5.00^{\circ }$	$5.05^{\circ }$	$5.10^{\circ }$
Trials	5	7	10	12	15	12	10	7	5
***Upper line fixed***									
Lower line length	$5.2^{\circ }$	$5.15^{\circ }$	$5.10^{\circ }$	$5.075^{\circ }$	$5.05^{\circ }$	$5.025^{\circ }$	$5.00^{\circ }$	$4.95^{\circ }$	$4.90^{\circ }$
Trials	5	7	10	12	15	12	10	7	5
